# Reducing the Bias of Norm Scores in Non-Representative Samples: Weighting as an Adjunct to Continuous Norming Methods

**DOI:** 10.1177/10731911231153832

**Published:** 2023-02-16

**Authors:** Sebastian Gary, Alexandra Lenhard, Wolfgang Lenhard, David S. Herzberg

**Affiliations:** 1 Psychometrica, Bavaria, Germany; 2 University of Würzburg, Germany; 3 Western Psychological Services, Torrance, CA, USA

**Keywords:** regression-based norming, continuous norming, weighted ranking, test development, representativeness, raking

## Abstract

We investigated whether the accuracy of normed test scores derived from non-demographically representative samples can be improved by combining continuous norming methods with compensatory weighting of test results. To this end, we introduce *Raking*, a method from social sciences, to psychometrics. In a simulated reference population, we modeled a latent cognitive ability with a typical developmental gradient, along with three demographic variables that were correlated to varying degrees with the latent ability. We simulated five additional populations representing patterns of non-representativeness that might be encountered in the real world. We subsequently drew smaller normative samples from each population and used an one-parameter logistic Item Response Theory (IRT) model to generate simulated test results for each individual. Using these simulated data, we applied norming techniques, both with and without compensatory weighting. Weighting reduced the bias of the norm scores when the degree of non-representativeness was moderate, with only a small risk of generating new biases.

In the development of norm-referenced psychometric tests, demographically representative samples provide the foundation for valid norm scores. An initial task for the test developer is to identify those demographic variables that correlate most strongly with the construct to be measured by the test. These variables typically include age, gender, race/ethnicity, education level, and/or socioeconomic status. When measuring a developing cognitive ability, age is the most important variable. Age has a stronger effect on test scores than the other variables, especially when testing children and adolescents. Because of this, Wechsler (1939, Chap. 3) recommended that same-age reference populations be used when norming tests of intelligence and achievement.

Consequently, normative samples must be demographically representative not just over the entire age range of the test but also within smaller age groups containing individuals who are at similar stages of development. Moreover, the normative samples for each individual age group must be large enough to allow the computation of reliable norms. These requirements increase the size of the entire normative sample (along with its cost and the time needed to collect it). To mitigate the need for larger samples, advanced mathematical methods have been developed to model the continuous relationship between raw and normed scores across age (e.g., Cole, 1988; Cole & Green, 1992; A. Lenhard et al., 2018a, 2018b, 2019; W. Lenhard & Lenhard, 2021; Oosterhuis, 2017; Oosterhuis et al., 2016; Stasinopoulos et al., 2018; Voncken et al., 2019; Zachary & Gorsuch, 1985).

Some norm-referenced measures additionally require consideration of variables other than age. For example, measures of body mass index (BMI) need separate norms for males and females because the optimal BMI for females is lower than that for males (Sang-Wook et al., 2015). In other instances, it may be counterproductive to provide separate norms for the different levels of a demographic variable. For example, some studies show that girls have higher reading skills than boys (e.g., W. Lenhard et al., 2017; Price-Mohr & Price, 2017). However, if a reading test is intended to identify those children who need additional support—for example, children at the lowest decile of reading performance—then the use of gender-specific norms might result in biased outcomes. With gender-specific norms, some girls might be identified as needing additional support, although they perform better on the test than boys who are not identified as needing educational support.

## Addressing Demographic Imbalances: Stratification and Post-stratification

Besides creating separate norms for specific demographic subgroups, several options exist for dealing with demographic variables that are correlated with the latent ability being measured. An obvious course is to increase the size of the normative sample. As the size of a randomly drawn sample increases, the distribution of the demographic variables in this sample increasingly approximates the distribution in the reference population. However, cost and time constraints usually limit the size of the sample available for norming. A second approach is stratification, in which random sampling is conducted independently within homogeneous categories, or strata, defined by the demographic variables (e.g., males, females). The goal is to have the category proportions in the normative sample match, as closely as possible, the proportions in the reference population. For example, if census data indicate that the reference population is composed of 50% males and 50% females,^1^ then the researcher would sample males and females independently to match those proportions in the normative sample.

However, it is not always possible to replicate population distributions through stratified random sampling. One can randomly delete cases from overrepresented strata, but researchers are understandably reluctant to discard data. An alternative is to apply weighting multipliers, or weights, to the data of individuals in the misrepresented strata. For example, if a sample consists of 100 males and 50 females, a weighting multiplier of 2 could be applied to the data obtained from females. Each test score from a female would then be treated as if two females had obtained such a result. A weight *w*_k_, assigned to an observation *x*_i_ in subsample *k*, thus indicates the number of individuals that this single observation represents. The weights must therefore be calculated so that the proportion

$$p_{k}=\frac{w_{k}\cdot n_{k}}{\sum_{l}w_{l}\cdot n_{l}},$$

corresponds to the proportion of stratum k in the reference population (with *n_k_* = size of subsample *k* in the normative sample). This weighting procedure is referred to as post-stratification (Little, 1993; Lumley, 2011, Chap. 7; Park et al., 2004).

Recently, Kennedy and Gelman (2021) recommended the use of multilevel regression combined with post-stratification to correct for non-representative samples in studies of psychological intervention. The authors suggest that weights can be used to adjust the means of non-representative samples to facilitate statistical comparisons among samples. However, in constructing test norms from non-representative samples, the application of weights is more complicated because the norming process involves modeling both population means and percentile ranks.

It is straightforward to take weights into account when calculating percentiles. As described earlier, each test result is treated as if obtained by w_k_ individuals. But this simple calculation runs the risk of introducing its own bias into the raw-to-norm-score relationship, especially at the tails of the raw-score distributions. This risk occurs because the weights do not change the variance of the distribution of raw scores within demographic subgroups as would be the case if more individuals were added to the subgroups. The potential distortion of the variance of the raw score distributions increases with the magnitude of the weights themselves. The risk for bias also *increases* as the number of individuals in a subgroup *decreases* (as is expected at the tails of the raw-score distributions). Consequently, the usefulness of weighting as a corrective procedure tends to diminish as the distributions of demographic variables in the normative sample become increasingly divergent from those in the reference population.

Because the potential distortions associated with weighting are most prominent at the tails of the raw score distributions, they can disproportionately affect the raw-to-norm-score relationships for individuals of very high and/or very low ability. Unfortunately, these extreme ability ranges are the ones where precise norm scores are most needed because the primary clinical applications of psychometric tests are to help diagnose disabilities, or, alternatively, to identify gifted individuals.

As noted earlier, post-stratification is a method for dealing with normative samples that are not representative, in terms of the distributions of demographic variables, of the reference populations from which they are drawn. An additional complicating factor is that the common demographic variables of gender, socioeconomic status (SES), race/ethnicity, and geographic region are often interrelated in terms of the effects they may have on test performance. For example, areas with lower household incomes often have higher proportions of non-white inhabitants. Because of such interactions, the most accurate approach to stratification is to consider not only the marginal distributions of the demographic variables but also their cross-classifications or joint distributions. In a complete crossing of the four variables mentioned earlier, for instance, an individual could be classified as “female, low SES, white, west region.”

There are several practical difficulties with stratification based on the joint distributions of demographic variables. For one, census data are often available only for single demographic variables considered independent of each other and not for the cross-classified categories of multiple variables. In addition, in one possible cross-classification of gender, SES, race/ethnicity, and region, 192 joint cells (2 × 4 × 6 × 4) are created, some of which require only a few individuals to meet census proportions. Collecting a sample that meets these exacting specifications becomes a costly, lengthy process. In fact, with typical sample sizes of 100 cases per age year in tests of cognitive ability (e.g., Kaufman & Kaufman, 2004; Wechsler, 2008, 2014), it is not possible to replicate the census proportions in every cross-classified cell because some of the joint percentages specify less than a single individual in a cell.

## Raking

The raking procedure (Ireland & Kullback, 1968; Kalton & Flores-Cervantes, 2003) is an approach to post-stratification that attempts to mitigate the practical challenges of sampling based on a complete crossing of demographic variables. Raking does not draw on the explicit joint distributions associated with all possible cross-classifications. Instead, the post-stratification weights are determined in an iterative process based on the marginal distributions of each demographic variable. That is, the weights assigned to the demographic categories are adjusted successively and, if necessary, repeatedly until they no longer change. The procedure is termed “raking” because it is analogous to smoothing out the soil in a garden bed by repeatedly raking in different directions. Studies have shown that the raking procedure is convergent and delivers optimal asymptotically normal estimates for the joint probabilities associated with a complete crossing of demographic variables (e.g., Ireland & Kullback, 1968).

Although widely employed to correct for lack of representativeness in political polls (Kalton & Flores-Cervantes, 2003), raking apparently has not been used in the norming of psychometric tests, perhaps because it could introduce error into the raw-to-norm-score relationships. As discussed earlier, demographic variables may interact with one another in their effects on test scores, creating the need to consider the joint distributions of such variables in developing norms. Because raking operates only on the marginal distributions (i.e., it considers only the “main effects” of demographic variables on test scores), it may magnify sources of error that stem from the interactions of these variables. However, these potential risks remain at the level of speculation because, to our knowledge, the effect of raking on the accuracy of norm scores has never been studied.

## Effects of Continuous Norming on Non-Representative Samples

Continuous norming methods offer the advantage of using the properties of the entire normative sample to correct local sampling errors in smaller subsamples (e.g., age strata). Consequently, continuous norming methods may offer at least a partial remedy to distortions caused by a lack of demographic representativeness in single age groups.

There have also been attempts to minimize systematic deviations of representativeness in normative data by combining parametric continuous norming methods with other statistical procedures. For example, Voncken et al. (2020) used Bayesian Gaussian distributional regression to align the distributions of newly collected normative data with prior information, more specifically, with previous normative data of the same test. In their simulation study, this method proved to be successful if the prior information was not biased itself. This method of course requires that a previous norm sample is available. And if it is available, it will be difficult to determine whether it is (still) representative of the reference population. Moreover, new normative data are usually collected precisely because the test has been revised or because one suspects that the distribution of the measured variable may have changed in the population. In practice, the use of prior information will therefore be restricted to very specific test development scenarios.

Another continuous norming approach is the semi-parametric continuous norming approach (SCN), first suggested by A. Lenhard and colleagues (A. Lenhard et al., 2018b, 2019; W. Lenhard & Lenhard, 2021). This method has been shown to yield accurate norm scores with several non-optimal types of normative samples. One advantage of SCN is that it does not make specific assumptions about distribution parameters and therefore can be applied to raw score distributions that are skewed or that show floor and/or ceiling effects.

Unlike parametric continuous norming approaches (e.g., Stasinopoulos et al., 2019; Voncken et al., 2019), SCN (as implemented in the cNORM package in R, A. Lenhard et al., 2018a) *does not* rely on splines to model the trajectories of percentile ranks across age groups but on simple polynomial regression. Therefore, the SCN approach is quite stiff, that is, the course of the curve is less influenced by individual data points compared with spline-based methods. As a result, these trajectories are relatively robust against single erroneous data points compared with spline-based regression. As noted earlier, this feature of SCN modeling tends to reduce the influence of error variance in local age groups, including that caused by age-specific lack of demographic representativeness. Therefore, SCN does not only produce less norm-score bias than methods that determine raw-to-norm-score mapping separately for each age group (W. Lenhard & Lenhard, 2021), but it also performs better than parametric continuous norming approaches when applied to normative samples with the typical sample size of 100 per age cohort, independent of the skewness of the raw score distributions (A. Lenhard et al., 2019).

## Goals of the Current Simulation Study

As described earlier, it has already been demonstrated that the SCN approach is very successful in smoothing out sampling errors that occur only in specific age groups. But it certainly cannot compensate for missing representativeness that systematically affects the entire normative sample. Raking, in turn, is a weighting procedure that seems to be optimally suited to compensate for a systematic lack of representativeness affecting the entire sample. But it cannot compensate for unsystematic error in single age groups caused by random sampling with a limited sample size. We therefore basically assumed that the combination of both methods would lead to an even greater improvement in the quality of norm scores because both types of errors described earlier are taken into account. But to date, no research has investigated whether the combination of both methods (in the following referred to as *weighted continuous norming*, or WCN; implemented in the R package cNORM v.3.0.2, A. Lenhard et al., 2018a) does in fact improve the accuracy of norm scores, compared with SCN alone. From a theoretical perspective, this should clearly be the case. However, there is also a risk that the mathematical transformations wrought by SCN and raking might interact in a way that *increases* the bias of norm scores, at least within certain ability ranges.

The goal of the current study, therefore, was to evaluate the benefits and risks of applying WCN to non-representative normative samples. To this end, we simulated normative data with different types and degrees of deviations from representativeness, applied both SCN and WCN to these data, and subsequently compared the results.

For the current study, our hypotheses were as follows:

**Hypothesis 1:** We expected a main effect of norming method, such that WCN would lead to less-biased estimates of the norm scores than SCN, where “bias” is quantified in terms of root mean square error (RMSE) and mean signed difference (MSD).
**Hypothesis 2:** We expected an interaction between norming method and the degree of non-representativeness of the input data. Specifically, we expected that as the non-representativeness of the normative sample increased, norm-score bias would increase for both methods, but that the increase in bias would be smaller for WCN than for SCN.
**Hypothesis 3:** We expected that the simple effect of WCN in reducing norm-score bias would vary depending on person location on the cognitive variable. Specifically, we expected that WCN would be less effective at reducing bias at the tails of the cognitive ability distribution than in the central part of that distribution.

## Method

### Overview

To answer the research questions, we conducted a norming procedure on a measure of a simulated cognitive ability that increases with age (cf. next section). Furthermore, we modeled the effects of three simulated demographic variables on the cognitive measure. For convenience, we labeled the simulated demographic variables as “education,”“ethnicity,” and “geographic region.” We modeled education so that it would have a stronger effect on the cognitive measure than ethnicity or region.

To provide input for the norming procedure, we generated six simulated population-level data sets: a reference population that embodied the benchmark distributions of the three demographic variables and five non-representative populations in which the distribution of these demographic variables differed from the reference population. Each of these populations had six equal-sized age cohorts. Table 1 summarizes the differences among the six simulated populations. Because raking incorporates only marginal distributions, we expected it to have little effect in Populations 5 (biased joint probabilities) and 6 (clustered sampling). In these two populations, non-representativeness occurs only at the level of joint distributions (cross-classifications) and not at the level of marginal distributions.

**Table 1:** Simulated Populations for Norming Input

No.	Label	Description	Hypothesized effects on distribution of cognitive ability variable
1	Reference	Benchmark distributions of demographic variables; the standard of comparison for describing the “representativeness” of the other simulated populations.	Not applicable (benchmark population).
2	Mild underrepresentation of high education	Lower proportion of high-education individuals, higher proportion of low-education individuals, than Population 1.	Both mean and variance affected.
3	Moderate underrepresentation of high education	The pattern of divergence of education proportions is similar to population 2, but the degree of non-representativeness is greater.	Both mean and variance affected.
4	Underrepresentation of both low and high education	Both tails of the education distribution have lower proportions than Population 1.	Only variance affected.
5	Biased joint distributions	Marginal distributions of demographic variables match population 1; joint distributions (cross-classifications) do not match population 1. The pattern of non- representativeness alternates from over- to underrepresented across the 27 (3 × 3 × 3) joint distributions.	Only variance affected. (Effects on mean too small to be of practical relevance.)
6	Clustered distributions	Marginal and joint distributions of demographic variables match population 1, but only when averaged across all six age cohorts. Within each age cohort, two-thirds of the joint distribution cells contain no data.	Variance affected in each separate age group. Overall variance and mean not affected.

In more detail, our simulation (data and R syntax available via https://osf.io/bwcre/) proceeded through the following steps:

1. Modeling a latent cognitive ability with a typical age-related growth curve.
2. Generating data sets for the reference population and five additional simulated populations.
3. Drawing normative samples from each simulated population.
4. Generating simulated raw scores for a test of the cognitive ability.
5. Applying WCN and SCN to the raw scores from the normative samples.
6. Generating norm scores based on the reference population, as a standard of comparison.
7. Comparison of norm scores determined with biased population and applying SCN versus WCN with the representative norms

### Modeling Cognitive Ability

To provide a basis for a modeled cognitive ability that develops with age, we envisioned a reference population divided into six age cohorts, spanning 1 year each. We conceptualized a cognitive ability that increases in each successive age group as is typical with cognitive development during childhood. We further specified that this cognitive ability is influenced by the three demographic variables, each of which has three categories, namely, education (low, medium, high), ethnicity (native, mixed, non-native), and region (south, east, northwest). In broad terms, therefore, our model states that cognitive ability is a function of age and the three demographic variables.

We operationalized the effect of the demographic variables on cognitive ability by assigning three levels of mean cognitive ability (below average, average, above average) to the three categories of each demographic variable, according to the matrix shown in Table 2. Importantly, this mapping of ability level to demographic category remains constant in all study conditions. Thus, by changing the distributions of demographic categories across simulated populations, we simultaneously manipulate the distributions of cognitive ability. We then specified benchmark distributions for the demographic variables that would be enacted in the simulated reference population data set. The benchmark demographic distributions must be understood in terms of a complete cross-classification of the three demographic variables, which yields a 27-cell matrix with a 3 (low, medium, high education) × 3 (native, mixed, non-native ethnicity) × 3 (south, east, northwest region) structure. Table 3 follows this structure in specifying the benchmark demographic distributions.

**Table 2:** Assignment of Cognitive Ability Levels to Demographic Categories

Demographic variable	Below-average ability	Average ability	Above-average ability
Education	Low	Medium	High
Ethnicity	Native	Mixed	Non-native
Region	South	East	Northwest

**Table 3:** Distributions of Demographic Variables, by Category, in the Reference Population

Ethnicity	Region	Low education 40%	Medium education 20%	High education 40%
Ethnicity: native 30%	region: south 60%	7.2	3.6	7.2
	region: east 20%	2.4	1.2	2.4
	region: north-west 20%	2.4	1.2	2.4
Ethnicity: mixed 40%	region: south 60%	9.6	4.8	9.6
	region: east 20%	3.2	1.6	3.2
	region: north-west 20%	3.2	1.6	3.2
Ethnicity: Non-native 30%	region: south 60%	7.2	3.6	7.2
	region: east 20%	2.4	1.2	2.4
	region: north-west 20%	2.4	1.2	2.4

*Note.* The benchmark marginal distributions of the demographic variables are shown in the table margins (for education and ethnicity) and in the left-most column of the nested rows (for the region). The joint distributions for the complete cross-classification of the three variables are shown in the table cells.

The structure of Table 3 provides a basis for understanding the demographic manipulations that were applied to create five additional simulated populations. As described previously, each cell of the table corresponds to a certain mean cognitive ability level, which is determined by the demographic cross-classification of that cell. Thus, referring to Table 2, the cell in Table 3 with the highest mean cognitive ability is high education, non-native ethnicity, and northwest region, which appears in the lower-right corner of the table, constituting 2.4% of the reference population. Conversely, the cell with the lowest mean cognitive ability is low education, native ethnicity, and south region, which appears in the upper-left corner of Table 3, constituting 7.2% of the reference population.

Within the reference population, each row of data includes age, level of cognitive ability, and classifications on education, ethnicity, and region. The values for the demographic classifications are assigned according to the percentages in Table 3. The row-wise values of cognitive ability are based on a distinct mean value for each cell^2^ of Table 3. This cell-wise mean is calculated by the following polynomial equation:

(1) $$\begin{array}{c} M\left(\mathrm{age},\mathrm{education},\mathrm{ethnicity},\mathrm{region}\right)=\\ \;-1.5\cdot \mathrm{education}-0.25\cdot \mathrm{ethnicity}-0.1\cdot \\ \;\mathrm{region}-0.05\cdot \mathrm{ethnicity}\cdot \mathrm{region}+1.2\cdot \mathrm{age}\\ \;-0.06\cdot \mathrm{ag}e^{2}+0.0001\cdot \mathrm{ag}e^{4}. \end{array}$$

As a result of this equation, each demographic variable exerts a different effect on cognitive ability: education correlates at *r* = −.78 with cognitive ability (large effect), ethnicity with *r* = −.54 (medium effect), and region with *r* = −.31 (small effect).

**Figure 1.**
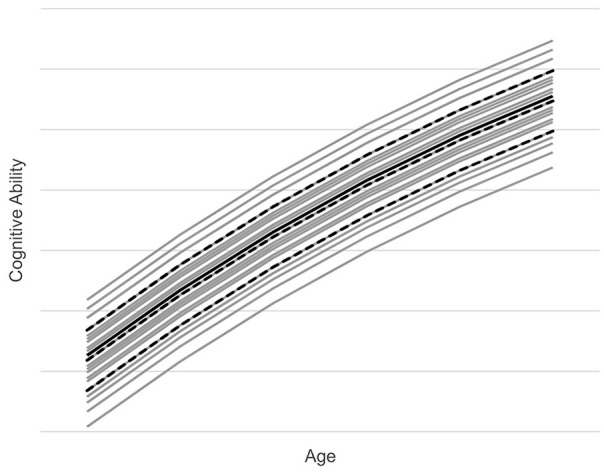
Modeled Cognitive Ability in Reference Population.

Figure 1 provides a graphic depiction of the modeled cognitive ability in the reference population. The figure shows mean cognitive ability increasing across the six age cohorts. The solid black line represents the reference population mean. The dashed black lines represent the marginal mean cognitive abilities for the low, medium, and high categories of education, the demographic variable with the largest effect on cognitive ability. The gray lines represent the mean cognitive abilities associated with the 27 demographic cross-classifications. The highest gray line is high education, non-native ethnicity, northwest region, the cell of Table 3 with the highest mean cognitive ability. The lowest gray line is low education, native ethnicity, and south region, the cell of Table 3 with the lowest mean cognitive ability.

### Generation of Simulated Population Data Sets and Normative Samples

To generate the reference population data set, we drew 24 million pairs of random numbers (4 million per age cohort), each pair representing one individual. The reference population size is roughly based on the number of persons in the United States. The first random number was uniformly distributed between 0 and 6 and represented age in years. The second number was normally distributed with *M* = 0 and *SD* = 1 within each of the six age groups and represented the cognitive ability of the individual with respect to other individuals of the same age. This random number was converted into the specific cognitive ability value for an individual by adding the mean cognitive ability for that individual’s demographic cross-classification status (see Table 3 and Formula 1). In addition, we *z*-standardized each cognitive ability value ( $x_{1}$ ), using the reference population mean μ and standard deviation σ in Equation 2,

(2) $$\theta_{\mathrm{pop}}=\frac{x_{1}-\mu }{\sigma },$$

where θ_pop_ represents an individual’s location on the cognitive ability variable with respect to the entire reference population.

As noted previously, each individual in the reference population was assigned values on the demographic variables such that marginal and joint distributions of these variables would match the distributions shown in Table 3. We then generated five additional simulated population data sets, using the same method described at the outset of this section (Table 1). These additional simulated populations represented various violations of demographic representativeness that might be encountered in collecting normative data for the development of a psychometric test. The distributions of the demographic variables in these five additional data sets differed from the reference population as follows:

- Simulated population 2: Mild underrepresentation of high education. The high education category was underrepresented (28% instead of 40%), and the low education category was overrepresented (52% instead of 40%). This manipulation affected both the mean and the variance of the cognitive ability variable.
- Simulated population 3: Moderate underrepresentation of high education. The pattern of misrepresentation was the same as in population 2, but the degree of misrepresentation was greater (high education was 20% instead of 40%; low education was 60% instead of 40%). This manipulation affected both the mean and the variance of the cognitive ability variable.
- Simulated population 4: Underrepresentation of both low and high education. Medium education was overrepresented (40% instead of 20%) and high and low education were underrepresented (30 % instead of 40 %). This manipulation attenuated the variance of the cognitive ability variable, but its mean was not affected.
- Simulated population 5: Biased joint distributions. The joint distributions of the demographic variables were varied from the reference percentages shown in Table 3 such that some of the joint cells were overrepresented while some were underrepresented, but the marginal distributions were identical to those in the reference population. This manipulation increased the overall variance of the cognitive ability variable, but its mean was only slightly affected.
- Simulated population 6: Clustered distributions. Within each age cohort, two-thirds of the 27 demographic cross-classification cells contained no data. In the remaining one-third of cells, the number of individuals was tripled. This manipulation was applied to different subsets of cells across age cohorts such that when cell proportions were summed across all age cohorts, the marginal and joint distributions of the demographic variables were identical to the reference population. This distribution condition was added to investigate the influence of clustered sampling as frequently applied in real test norming projects due to economic constraints. This manipulation either increased or attenuated the variance of the cognitive ability in each separate age group, but it did not affect the overall mean or variance.

In the five additional simulated population data sets, the cognitive variable was z-standardized using Equation 2. Importantly, the values of μ and σ were those from the reference data set (population 1), not from the data set whose values were being standardized. By using μ and σ of the reference population, the standardized variable reflects bias resulting from biased norm sampling. For example, using Equation 2 for simulated population 2 with μ and σ of the reference population resulted in standardized values for the cognitive ability with a mean value higher than 0, since subjects with high cognitive abilities were overrepresented with respect to the reference population. From each of the six simulated populations, we drew 100 random samples of 600 individuals (100 cases per age cohort). These samples served as input to the norming procedures.

### Simulation of Test Results

Using the one-parameter logistic (1-PL) model, we simulated a 31-item test to generate test results for each individual in the normative samples. The 31 item difficulties (δ) were drawn randomly from a uniform distribution ranging from −3 and +3. The set of item difficulties covered a range of about 3.7 standard deviations (*M* = −0.04, *SD* = 1.64), therefore spanning a wide range of latent ability. The probability *p*_k, *i*_ that an individual *k* with the *z*-standardized latent ability ${\theta_{\mathrm{pop}}}_{k}$ succeeded on item *i*, with difficulty δ_*i*_, was given by the following 1-PL equation:

(3) $$p_{k,i}\left(x_{i}=1|\theta_{\mathrm{po}p_{k}},\delta_{i}\right)=\frac{\exp\left(\theta_{\mathrm{po}p_{k}}-\delta_{i}\right)}{1+\exp\left(\theta_{\mathrm{po}p_{k}}-\delta_{i}\right)}.$$

For every individual *k* and item *i* a uniformly distributed random number between 0 and 1 was drawn and compared to *p_k_*,_*i*_. If *p_k_*,_*i*_ exceeded the random number, the item was scored 1, otherwise it was scored 0. Finally, each individual’s scores on all 31 items were summed to yield a raw total score on the simulated test.

### Application of Weighted and Unweighted Norming Procedures

For each raw score in the normative samples, we applied WCN and SCN to generate IQ-type standard scores (*M* = 100, *SD* = 15) for each norming method. These scores were labeled IQ_WCN_ and IQ_SCN_. For both WCN and SCN, these IQ scores were calculated with *cNORM*, an R package that employs continuous norming (A. Lenhard et al., 2018a). Weights were not used for SCN.

To calculate the weights for WCN, we used the *rake* function from *survey* (Lumley, 2011), an R package that implements the raking procedure described earlier in this article. In addition, we standardized the weights to make them easier to interpret. We divided each weight by the smallest weight in the respective norm sample, thereby setting the weight of the most overrepresented group in the sample to 1.

Using weights required modifications to the standard cNORM functions. In WCN, weights are applied initially in the ranking procedure, where each raw score is assigned a percentile rank. Because of the high number of ties, the average rank was used for further processing, following the usual cNORM procedures (see A. Lenhard et al., 2019). In WCN, weights are also entered in cNORM’s regression-based modeling procedure. To perform the regression, cNORM draws on the *regsubsets* function of *leaps*, an R package (Lumley, 2017). *regsubsets* includes the capacity to process weights in the regression analysis.

### Generating Norm Scores From the Reference Population

To test the study hypotheses, we created a measure (IQ_best_), in the same metric as IQ_WCN_ and IQ_SCN_, which represented the “actual” person location on the cognitive ability variable. IQ_best_ was derived from the distribution of raw scores in the entire reference population (in contrast to IQ_WCN_ and IQ_SCN_, which were derived from the smaller normative samples). To compute IQ_best_, we generated raw scores on the 31-item simulated test for the 24 million individuals in the reference population, using the previously described method. We then partitioned the reference population by age, creating 365 equal-sized groups within each of the six age cohorts. Each of the resulting 2,190 age groups consisted of about 11,000 individuals with the same “birthday.” The raw scores were ranked and converted into IQ scores using rank-based inverse normal transformation within each age group. As a result, each row in the reference population data set included values for age, raw score, and IQ_best_.

#### Hypothesis Testing With RMSE and MSD

As noted earlier, we drew 100 normative samples from each of the six simulated population data sets (*N* = 600) and subjected them to both SCN and WCN. For each of the samples, we computed RMSE and MSD to compare IQ_best_ to IQ_WCN_ and IQ_SCN_, respectively.

RMSE is a summary measure of norming model error that includes both fixed and variable error components (W. Lenhard & Lenhard, 2021). It was computed using the following formula:

(4) $$\mathrm{RMSE}=\sqrt{\frac{1}{n}\sum_{i=1}^{n}{\left(IQ_{.}-IQ_{\mathrm{best}}\right)}^{2}},$$

where n is the number of cases and IQ. stands for either IQ_WCN_ or IQ_SCN_. MSD is a measure of the tendency for a norming model to overestimate (MSD > 0) or underestimate (MSD < 0) the actual person location. The formula used to calculate the MSD was

(5) $$\mathrm{MSD}=\frac{1}{n}\sum_{i=1}^{n}\left(IQ_{.}-IQ_{\mathrm{best}}\right).$$

To be able to test Hypothesis 3, we divided the distributions of IQ_best_, IQ_WCN_, and IQ_SCN_ into 11 intervals of 7.5 IQ points each. RMSE and MSD were calculated separately for each of these intervals. Both RMSE and MSD are quantified in terms of IQ points.

In general, the analytic approach was to conduct 6 (simulated population) × 11 (IQ range) × 2 (norming method) mixed analyses of variance (ANOVAs) on both RMSE and MSD. The population was a between-groups factor, and IQ range and norming method were within-groups factors. Because of the high number of simulation cycles (i.e., *n* = 100 within each cell of the ANOVAs), statistical power was high, and therefore the level of significance was set to *p* = .01. The assumption of sphericity was tested, and, where indicated, the degrees of freedom were corrected. In addition, partial η^2^s were computed as measures of effect size. We further specified that, in the norm score comparisons of interest, differences of <1 IQ point were too small to have any practical relevance. We opted for this threshold, as IQ scores are usually rounded to integers in test manuals, and, therefore, differences of 1 IQ point or more represent the smallest detectable difference in the norm tables.

## Results

As indicated by Mauchly’s test, sphericity assumptions were generally violated both for RMSE and MSD. Therefore, degrees of freedom in all ANOVAs were corrected according to the Greenhouse-Geisser method. The two separate 6 × 11 × 2 mixed ANOVAs on RMSE and MSD yielded significant results for all main effects and interactions (*p* < .001). We focus here on the effects that are most salient for testing our hypotheses.

### Hypothesis 1: Main Effect of Norming Method

The first hypothesis proposed that WCN would yield lower levels of norm score bias than SCN. This hypothesis was supported by tests of the main effects of norming method, RMSE: *F*(1, 594) = 94.93, *p* < .001, η^2^ = .14, MSD: *F*(1, 594) = 3397.28, *p* < .001, η^2^ = .85. RMSE was smaller for WCN (*M* = 2.18, *SE* = .02) than for SCN (*M* = 2.36, *SE* = .02). The same was true for MSD (SCN: *M* = 0.74, *SE* = .03; WCN: *M* = -0.24, *SE* = .03).

**Figure 2.**
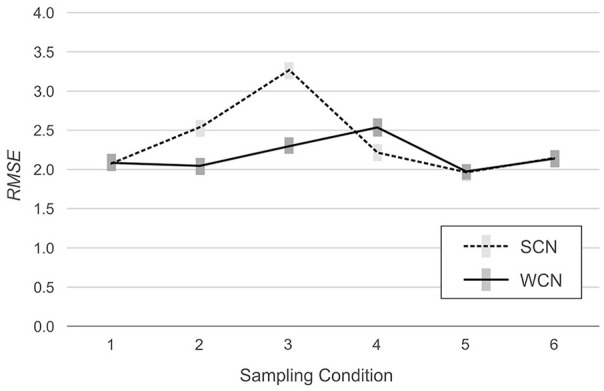
RMSE Across Simulated Populations, With (WCN) or Without (SCN) Weighting. *Note.* The gray rectangles represent 95% confidence intervals. RMSE = root mean square error; WCN = weighted continuous norming; SCN = semi-parametric continuous norming.

**Figure 3.**
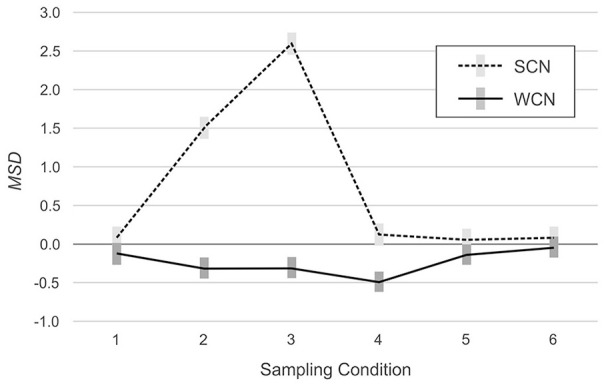
MSD Across Simulated Populations With (WCN) or Without (SCN) Weighting. *Note.* The gray rectangles represent 95% confidence intervals. MSD = mean signed difference; WCN = weighted continuous norming; SCN = semi-parametric continuous norming.

However, the analysis also detected significant interactions between norming method and simulated population, indicating that the effects of weighting on norm-score bias varied among the simulated populations, RMSE: *F*(5, 594) = 98.98, *p* < .001, η^2^ = .45, MSD: *F*(5, 594) = 764.77, *p* < .001, η^2^ = .87. As can be seen in Figure 2 (RMSE) and Figure 3 (MSD), in two of the six simulated populations, weighting reduced bias in the normed scores. In Populations 2 (mild underrepresentation of high education) and 3 (moderate underrepresentation of high education), RMSE was lower with WCN than with SCN, by an average of 0.48 IQ points and 0.97 IQ points, respectively. In Populations 1 (reference), 5 (biased joint probabilities), and 6 (clustered sampling), the difference in RMSE between WCN and SCN approximated zero. In Population 4 (underrepresentation of both low and high education), WCN returned a higher average RMSE than SCN, but the difference of 0.32 IQ points was below the threshold of practical relevance.

The analysis of the MSD yielded similar results. In Populations 2 (mild underrepresentation of high education) and 3 (moderate underrepresentation of high education), MSD was closer to the ideal value of zero for WCN (Populations 2 and 3: −0.32 IQ points) than for SCN (Population 2: 1.51 IQ points; Population 3: 2.59 IQ points). In Populations 1 (reference), 5 (biased joint probabilities), and 6 (clustered sampling), MSD approximated zero, regardless of the norming method. In Population 4 (underrepresentation of both low and high education), MSD deviated more from zero for WCN (−0.49 IQ points) than for SCN (0.13 IQ points). As with *RSME*, these latter differences did not meet the criterion for practical significance.

### Hypothesis 2: Interaction Between Norming Method and Degree of Non-Representativeness

Hypothesis 2 specified that as the non-representativeness of the normative samples increased, the norm-score bias would increase for both methods but that the increase in bias would be smaller for WCN than for SCN. To address this hypothesis, we compared Populations 2 and 3. Both populations were characterized by underrepresentation of the high education group, but the magnitude of underrepresentation was greater in Population 3 than in Population 2. Therefore, we performed two additional ANOVAs that limited the levels of the between-groups factor to Populations 2 and 3. Both analyses yielded a significant interaction between norming method and simulated population, RMSE: *F*(1, 198) = 26.01, *p* < .001, η^2^ = .12, MSD: *F*(1, 198) = 242.36, *p* < .001, η^2^ = .55.

The results of these analyses are visualized in Figures 2 and 3. The plots show the interaction: The RMSE is greater in magnitude in Population 3 (moderate underrepresentation) than in Population 2 (mild underrepresentation), for both norming methods, but the magnitude of the increase is greater for SCN (.74 IQ points) than for WCN (.25 IQ points). With regard to MSD, the benefits of WCN were even more pronounced. Considering WCN in isolation, average MSD was approximately equal for both populations (−0.32 IQ points). By contrast, with SCN, MSD in Population 3 was 1.09 IQ points higher than in Population 2.

### Hypothesis 3: Effectiveness of WCN Depends on Person Location

Hypothesis 3 proposed that WCN would be less effective at reducing bias at the tails of the cognitive ability distribution than in the central part of that distribution. We tested this hypothesis with two analytic approaches. First, we conducted 11 × 2 ANOVAs with person location and norming method (WCN vs. SCN) as within factors, and RMSE and MSD as separate dependent variables. Thus, a total of 12 different ANOVAs were calculated in this first analytic approach (two for each of the six sampling conditions).

**Figure 4.**
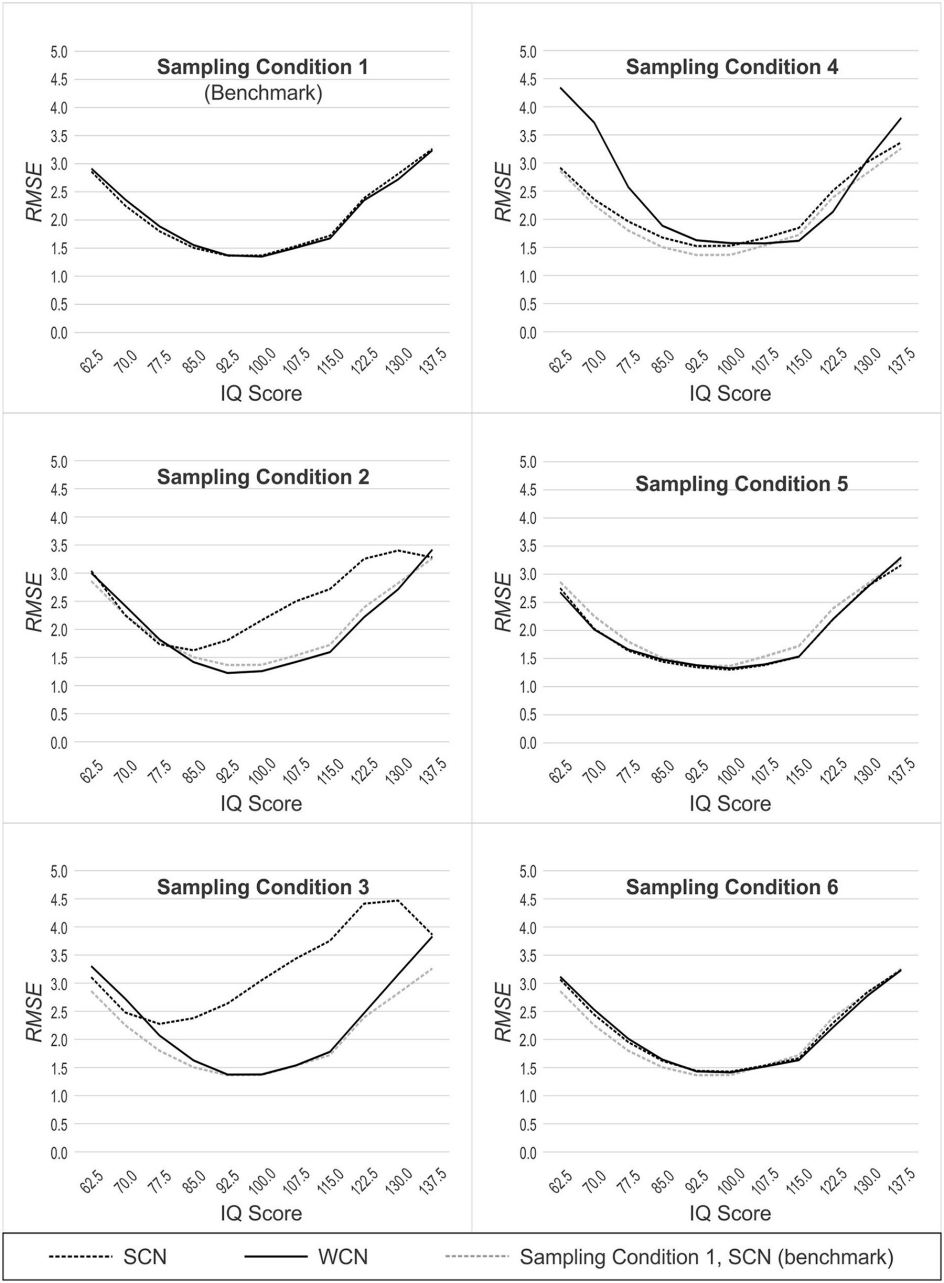
RMSE Across Simulated Populations, With (WCN) or Without (SCN) Weighting, as a Function of Person Location. *Note.* The dotted gray line represents SCN with norm samples drawn from population 1 (benchmark). RMSE = root mean square error; WCN = weighted continuous norming; SCN = semi-parametric continuous norming.

In Populations 2, 3, 4, 5 and 6 (which yield demographically non-representative normative samples, as described earlier), we additionally compared the performance of WCN to SCN in Population 1 (which yields demographically representative normative samples). SCN in Population 1, therefore, represents a benchmark condition, against which the performance of WCN in the other non-representative populations can be measured. For this second analytic approach, we also used 11 × 2 ANOVAs, but this time with norming condition (WCN with biased normative sample vs. SCN with unbiased normative sample) as a between-group factor. The results of these analyses are illustrated in Figures 4 (RMSE) and 5 (MSD). Because of the large number of comparisons, we report effects only if at least one of the differences within an analysis exceeded 0.5 IQ points.

#### Population 1: Reference

**Figure 5.**
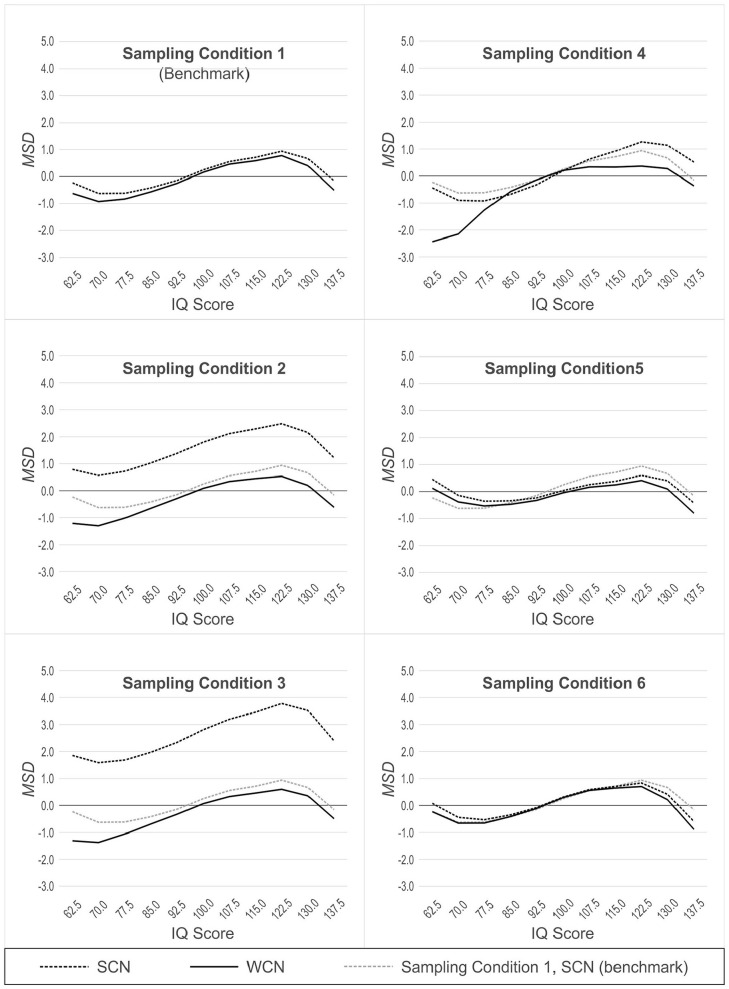
MSD Across Simulated Populations, With (WCN) or Without (SCN) Weighting, as a Function of Person Location. *Note.* The dotted gray line represents SCN with norm samples drawn from population 1 (benchmark). MSD = mean signed difference; WCN = weighted continuous norming; SCN = semi-parametric continuous norming.

In normative samples drawn from the reference population, the ANOVAs for RMSE and MSD both yielded a significant main effect of person location, RMSE: *F*(2.68, 264.87) = 70.68, *p* < .001, η^2^ = .42, MSD: *F*(2.34, 231.65) = 35.54, *p* < .001, η^2^ = .26. In general, RMSE increased as person location moved toward either tail of the distribution, away from the average IQ of 100. This effect, also seen in the other simulated populations, is visualized as a parabolic shape in Figure 4. By contrast, in the analysis with MSD, the main effect of a person location is visualized as a sinusoidal pattern (see Figure 5 and discussion section below). This effect of person location on norming bias is a previously reported feature of continuous norming procedures (cf. A. Lenhard et al., 2019). As such, this effect is not directly relevant to the question of whether weighting, per se, reduces norm-score bias due to non-representative sampling. What is important to note (and is readily seen in Figures 4 and 5) is that WCN and SCN perform equally well, in terms of error measures, when processing normative samples drawn from a demographically representative, reference population. This makes intuitive sense because with representative samples, there are no cell-wise departures from expected demographic proportions to which weights could be applied to correct for bias in the norming process.

#### Population 2: Mild Underrepresentation of High Education

In samples drawn from Population 2, we found a main effect of the norming method, RMSE: *F*(1, 99) = 74.94, *p* < .001, *η*^2^ = .43, MSD: *F*(1, 99) = 1,924.64, *p* < .001, η^2^ = .95. WCN was superior to SCN in reducing norm-score bias resulting from non-representative samples. With RMSE, we also observed an interaction between person location and norming method, *F*(2.72, 268.76) = 41.72, *p* < .001, η^2^ = .30. As shown in Figure 4, WCN reduced the error measure to a greater degree in the upper range of person location than in the lower range. In Population 2, individuals of higher education (and consequently, higher cognitive ability) are underrepresented. Thus, the interaction shows that WCN is correcting for norm-score bias at those person locations that are underrepresented in the normative samples.

In the comparison of WCN in Population 2 to the benchmark of SCN in Population 1, there was no main effect of the norming condition on RMSE. That is, even under the conditions of non-representativeness in Population 2, WCN did not differ from the benchmark on the error measure. This suggests that weighting successfully compensated for any norm-score bias due to demographic non-representativeness in Population 2 when that bias was measured by RMSE. The results differed for MSD, where we observed a main effect of norming condition, *F*(1, 198) = 15,37, *p* < .001, η^2^ = .07, and interaction between norming condition and person location, *F*(2.31, 456.93) = 2.91, *p* = .048, *η*^2^ = .01. These findings indicated that, with respect to MSD, WCN did not fully correct norm-score bias in samples from Population 2. However, this difference in MSD between WCN and the benchmark condition was generally rather small. Only at a very low person location of IQ 62.5, the difference (0.99 IQ points) approached the practically relevant threshold of 1 IQ point.

#### Population 3: Moderate Underrepresentation of High Education

For both error measures, the ANOVAs with normative samples drawn from population 3 yielded significant main effects of norming method, RMSE: *F*(1, 99) = 155.76, *p* < .001, η^2^ = .61, MSD: *F*(1, 99) = 2,707.76, *p* < .001, η^2^ = .97, and significant interactions between norming method and person location, RMSE: *F*(2.79, 276.13) = 71.89, *p* < .001, η^2^ = .42, MSD: *F*(2.63, 260.05) = 6.58, *p* = .001, η^2^ = .06. The analyses for Population 3 produced larger effect sizes than those for Population 2, mirroring the difference in representativeness between the two populations. This suggests that WCN exerts a larger corrective effect on the norm-score bias with normative samples that display greater deviations from demographic representativeness.

In comparing WCN in Population 3 to SCN in Population 1, we observed significant main effects of the norming condition, RMSE: *F*(1, 198) = 9.52, *p* = .002, η^2^ = .05, MSD: *F*(1, 198) = 12.52, *p* = .001, η^2^ = .06, and significant interactions between norming condition and person location, RMSE: *F*(2.81, 557.01) = 3.06, *p* = .031, η^2^ = .02, MSD: *F*(2.26, 447.76) = 3.28, *p* = .033, η^2^ = .02. In the normative samples drawn from Population 3, WCN yielded greater norming error than the benchmark within the low and high levels of person location. However, the differences were too small to be of practical relevance.

#### Population 4: Underrepresentation of Both Low and High Education

As with Populations 2 and 3, the analyses of normative samples drawn from Population 4 produced significant main effects of norming method, RMSE: *F*(1, 99) = 39.02, *p* < .001, η^2^ = .28, MSD: *F*(1, 99) = 164.63, *p* < .001, η^2^ = .62, and significant interactions between norming method and person location, RMSE: *F*(3.02, 299.01) = 42.43, *p* < .001, η^2^ = .30, MSD: *F*(2.53, 250.47) = 42.44, *p* = .001, *η*^2^ = .30. However, with Population 4, where both tails of the education distribution were underrepresented, WCN did not provide greater reduction of norm-score bias than SCN. The interactions revealed that at low levels of person location, RMSE was even greater for WCN than that for SCN (IQ 70.0|ΔRMSE| = 1.37 IQ points, IQ 62.5 (|ΔRMSE| = 1.43 IQ points). For MSD, the interaction between norming method and person location was more complex, with SCN providing a greater reduction of norm-score bias than WCN at low levels of person location (IQ 70.0:|ΔMSD| = 1.25 IQ points; IQ 62.5:|ΔMSD| = 2.00 IQ points). In the upper levels of person location, WCN narrowly outperformed SCN, but the differences were below the threshold of practical relevance.

In comparing WCN in Population 4 to SCN in Population 1, we found significant main effects of norming condition similar to those we just described when comparing WCN to SCN in population 4., RMSE: *F*(1, 198) = 38.46, *p* < .001, η^2^ = .16, MSD: *F*(1, 198) = 31.10, *p* < .001, η^2^ = .14. We also found similar interactions between norming condition and person location, RMSE: *F*(2.83, 560.75) = 22.19, *p* < .001, η^2^ = .10, MSD: *F*(2.55, 505.02) = 19.44, *p* < .001, η^2^ = .09. For both RMSE and MSD, WCN delivered significantly worse results than the benchmark condition at low person locations. For person locations of IQ 70 or lower, these differences exceeded the practically relevant threshold of 1 IQ point (IQ 70.0:|Δ RMSE| = 1.47 IQ points;|Δ MSD| = 1.52 IQ points; IQ 62.5:|Δ RMSE| = 1.48 IQ points;|Δ MSD| = 2.21 IQ points). For high-person locations, the results were again more complex, but the differences were generally too small to be of practical relevance in this ability range.

#### Population 5 (Biased Joint Distributions)

In samples drawn from population 5, we found no consistent effects demonstrating either superiority or inferiority of WCN compared with SCN. For RMSE, we observed a significant interaction between person location and norming method, *F*(3.11, 307.61) = 4.27, *p* = .005, η^2^ = .04. For MSD, we found a significant main effect of norming method, *F*(1, 99) = 51.18, *p* < .001, η^2^ = .34, and a significant interaction between norming method and person location, *F*(1.54, 152.20) = 11.21, *p* < .001, η^2^ = .10. But both interactions proved to be disordinal, that is, WCN performed better than SCN at some person locations and worse at others. Furthermore, all differences were much smaller than 1 IQ point and therefore of no practical relevance.

In the comparison of WCN in Population 5 to SCN in Population 1, we observed a significant interaction between norming condition and person location for MSD only, *F*(2.29, 453.98) = 6.25, *p* = .001, η^2^ = .03. But again, the interaction was disordinal and the differences were far too small to be of any practical relevance. Hence, the differences between WCN, SCN, and the benchmark (SCN in Population 1) were generally very small in samples drawn from Population 5. We will further elaborate on this result in the discussion section.

#### Population 6 (Clustered Distributions)

For Population 6, the comparison between WCN and SCN yielded no significant effects at all regarding RMSE. For MSD, the ANOVA returned a significant main effect of the norming method, *F*(1, 99) = 18.95, *p* < .001, η^2^ = .16, and a significant interaction between norming method and person location, *F*(1.65, 163.79) = 10.04, *p* < .001, η^2^ = .09. But as in population 5, the interaction was disordinal, that is, the effects of weighting were inconclusive with amelioration of the norm scores at some person locations but deterioration at others. Furthermore, the differences between WCN and SCN were even smaller than in population 5.

The comparison of WCN in Population 6 to the benchmark of SCN in Population 1 yielded no significant effects at all.

## Discussion

The present study examined whether compensatory weighting at the raw score level, when combined with semi-parametric continuous norming, would reduce bias in norm scores derived from demographically non-representative norm samples. To pursue this aim, we simulated six populations in which the distributions of demographic variables departed to various degrees from expected proportions. We modeled a latent cognitive ability, which we used as the input for a one-parameter logistic IRT model to create raw test scores. We drew normative samples from the six populations and generated IQ-type norm scores by applying WCN and SCN without weighting. We used RMSE and MSD as measures of norm-score bias.

Our first hypothesis proposed that when processing non-representative normative samples, WCN would produce less-biased norm scores than SCN. The predicted general advantage of WCN was most apparent in samples drawn from Populations 2 and 3 in which individuals with high levels of education were underrepresented. In samples drawn from Populations 5 (biased joint probabilities) and 6 (clustered sampling), WCN showed no benefit over SCN but neither did it degrade the quality of norm scores, relative to continuous norming without compensatory weighting. In Population 4 (underrepresentation of both low and high education), we found that WCN even led to a small increase in norm-score bias, but only at certain points in the range of cognitive ability.

In normative samples drawn from Population 1, which served as the standard of representativeness for the demographic variables, WCN demonstrated no advantage over SCN. This result is not surprising: WCN creates weights to compensate for departures from representativeness. Because Population 1 was the benchmark, in terms of demographic composition, random samples drawn from it were expected to be demographically representative.

Population 2 introduced deviations from the benchmark distribution of education, the demographic variable with the strongest effect on cognitive ability. Specifically, Level 1 (high education/high ability) was mildly underrepresented, and Level 3 (low education/low ability) was proportionately overrepresented. In samples drawn from Population 2, WCN yielded a greater reduction in norm-score bias than SCN, for both error measures, across the entire range of cognitive ability.

Population 3 presented a pattern of non-representativeness in education that was similar to that in Population 2 but greater in magnitude. The comparison of normative samples drawn from Populations 2 and 3 was relevant to testing our second hypothesis, which specified that as the non-representativeness of the normative sample increased, norm-score bias would increase for both methods but that the increase in bias would be smaller for WCN than for SCN. Our findings provided support for this hypothesis: With samples drawn from Population 3, the magnitude of norm-score bias for WCN was larger than it was in the Population 2 analyses, although WCN retained its superiority to SCN in terms of reducing this bias. As compared with the benchmark condition (i.e., SCN in the unbiased population 1), the increase in norming error associated with WCN in Population 3 depended on person location—it occurred at either extreme of the range of the cognitive ability variable but not at an average level. In no instances, however, did these increases in the error measures exceed 1 IQ point. Hence, even with this strong deviation from representativeness, WCN did a very good job at reducing the corresponding norm-score bias.

Population 4 embodied a further scenario of demographic non-representativeness, in which both tails of the education distribution were underrepresented, and the central part of the distribution was proportionately overrepresented. In terms of the average degree of misrepresentation across the three levels of education, Population 4 did not differ from Population 3. Where the effect of the demographic manipulation differs is on the raw score distributions. In Population 4, the manipulation attenuates the variance of the raw score distributions, because undersampling both tails of the education distribution results in an undersampling of the very high and low raw scores that reside in those ability levels. In addition, While in Population 3 the pattern of misrepresentation affects the mean of the raw score distribution, in Population 4, the mean is not affected, because there is an equal underrepresentation of both tails of the raw score distribution.

In normative samples drawn from Population 4, we observed that at certain levels of person location, WCN was less effective than SCN in reducing norm-score bias, which is consistent with our third hypothesis. Specifically, we found that the disparity between WCN and SCN increased at both tails of the cognitive ability distribution, with WCN showing the greatest magnitude of norming error at the lowest levels of person location.

To put this finding into context, consider how the raw score distribution of a demographic subgroup is affected differentially by adding additional individuals, as opposed to weighting the existing raw scores without increasing the sample size. Adding more individuals increases the variance of the raw score distribution, whereas weighting existing raw scores does not affect the variance. In Population 4, furthermore, the variance of the low- and high-ability groups was reduced by the pattern of underrepresentation, which results in fewer individuals in each of these groups. Therefore, weighting the raw scores of the underrepresented groups increases the influence of any sampling error that exists in the raw score distributions. This phenomenon may explain our finding that WCN resulted in greater norming error at the underrepresented, low levels of person location. By contrast, WCN did not yield increased norming error at the average levels of person location, where there are more observations present and a consequent reduction in sampling error. Our findings suggest that researchers should employ WCN with caution when processing normative samples where both low-performing and high-performing subgroups are substantially underrepresented.

In Population 5, the joint distributions of the demographic variables (resulting from a complete cross-classification of the three variables) were manipulated in a pattern of alternating over- and underrepresentation. This pattern was accomplished so that the marginal distributions of the variables closely approximated those of the reference population. Thus, Population 5 simulates a sampling scenario wherein demographic misrepresentation occurs at a level that is “beyond the reach” of cNORM’s raking method, which operates only on marginal distributions.

Under these conditions, WCN did not provide any improvement in the reduction of norm-score bias over SCN. However, our manipulation of the joint probabilities, as it turned out, did not strongly affect the means and variances of the raw score distributions. On one hand, this result leaves unanswered the question of how WCN might perform when misrepresentation at the level of joint probabilities does bias the parameters of the raw score distributions more severely. On the other hand, we would also like to emphasize here, that our aim was not necessarily to simulate deviations from representativeness as large as possible. Instead, we wanted to simulate different realistic scenarios and demonstrate the size of the respective effects, no matter whether these effects are large or small. With Population 5, we wanted to simulate a condition where the marginal distributions of the demographic variables satisfy representativeness, but the joint distributions do not. We sought to manipulate the distributions of the demographic variables in such a way that the resulting raw score distribution would deviate as much as possible from the unbiased distribution in Population 1. But with perfectly representative marginal probabilities, this is in fact virtually impossible (at least we did not succeed). What can we possibly learn from this specific condition? We can learn that one need not worry too much about the joint distributions of the demographic variables as long as the marginal distributions are in line with representativeness (which, by the way, is the principle of raking).

It is important to keep in mind, though, that in our simulation study, the three demographic variables were modeled so that education had the strongest relationship with cognitive ability and thus had more impact on norm score accuracy than ethnicity or region. Thus, our findings with Population 5 do not reflect the range of possible relationships between demographic factors and cognitive ability (e.g., other variables that are highly correlated with ability, or variables that interact with each other). In these alternate scenarios, misrepresentation in the joint distributions might possibly affect raw score means and variances more severely than was the case in Population 5 in this study. Later in this section, we provide guidance on how to address these scenarios in practice.

In Population 6 (clustered distributions), the distributions of the demographic variables were manipulated *within* each of the six age cohorts. This manipulation is best understood in comparison to Population 1, in which the marginal and joint probabilities of the entire population are replicated within each age cohort. In Population 6, by contrast, two-thirds of the joint distribution cells contained no data, meaning that the overall demographic distributions were *not* replicated *within* the age cohorts. However, the pattern of data deletion was such that the marginal and joint probabilities of the demographic variables, averaged over the entirety of the population (across all age cohorts), matched those of population 1.

In normative samples drawn from Population 6, the age-specific patterns of demographic non-representativeness affected the parameters of the raw score distributions within each age cohort. Raking per se cannot compensate for discrepancies of this nature because raking operates on marginal probabilities of the entire normative sample and not those within each age cohort. At first glance, it may seem counterintuitive to find, as we did, that neither WCN nor SCN yielded increases in norm-score bias when compared with the benchmark condition. We attribute this finding to the influence of the semi-parametric continuous norming method that underlies both WCN and SCN. As noted previously, this method models the raw-score–norm-score relationship as a function of age and person location using polynomial regression over the complete age range of the norm sample instead of computing the norm scores for each age cohort separately. Therefore, the previously mentioned stiffness of the method seems capable to reduce the effects of the varying age-specific violations of demographic non-representativeness.

### Implications for the Use of WCN in Test Norming

Our study showed that WCN reduces norm-score bias under certain patterns of non-representativeness of a demographic variable, where that variable is strongly correlated with the test score being normed. The pattern of results across the six simulated populations, however, suggested that even when a demographic variable has a strong effect on raw scores, it produces relatively small distortions in resulting norm scores. Even under conditions representing large departures from demographic representativeness, the differences in RMSE between norm scores derived using WCN and those from representative samples did not exceed 2 IQ points. With norming methods that generate norms independently for each age group, we would expect departures from demographic representativeness to cause greater levels of norm-score bias (W. Lenhard & Lenhard, 2021). These conventional norming methods lack the previously noted advantage of continuous norming, which can smooth out local effects of non-representativeness. Consistent with this view, we have demonstrated previously that with conventional norming per age group, RMSE is more than 3 times as high, on average, as with semi-parametric continuous norming, even with representative random samples (W. Lenhard & Lenhard, 2021). The selection of an appropriate norming method is therefore a critical prerequisite for accurate test norms, regardless of whether this procedure is used with or without weighting. By contrast, the size of the normative sample is less critical, if continuous norming is used. For example, we found that increasing the sample size from 100 to 250 per age group did not yield a significant reduction in RMSE, when continuous norming methods were used (A. Lenhard et al., 2019).

Clearly, the best practice is to prevent problems associated with non-representativeness in the first place, by collecting an adequately sized, demographically representative sample for norming. Post hoc weighting procedures are no substitute for a well-planned data collection effort that draws randomly from the general population. Care must also be taken to avoid oversampling from clinical settings, as this will bias the sample toward individuals of lower ability. The current study demonstrates the utility of weighting procedures in reducing norm-score error under conditions of mild-to-moderate non-representativeness of a demographic variable. Nevertheless, we also found that the ability of WCN to reduce norm-score bias was degraded when we reduced the marginal probability of the high level of education to 20% from the reference value of 40% (i.e., when the size of that subgroup was half that needed for a representative sample). Our work further shows that the effectiveness of weighting depends on the location of underrepresented demographic groups on the spectrum of person ability. With a typical cognitive ability that is normally distributed in the general population, random sampling will yield relatively small subgroups at either tail of the ability distribution. If these extreme subgroups are undersampled to begin with, any sampling error embodied in the raw score distributions will only be multiplied by the application of compensatory weights. This can lead to increased norm-score bias, as illustrated in our results. The remedy, of course, is to ensure that these low- and high-ability groups are represented in adequate numbers.

As described previously, the raking procedure used in this study operates only on the marginal distributions of the demographic variables. Census information on the joint distributions of the demographic variables (e.g., the expected probability for the joint category of low education/non-white ethnicity) is not always available. However, when that information is available, it can be incorporated into the raking procedures through a recoding process. For example, the crossing of two demographic variables, each of which has three categories, results in nine cross-classification cells. These classifications can be recoded into nine levels of a single dummy variable. The expected joint probabilities of the cross-classified cells thereby become the expected marginal probabilities of the dummy variable. The risk in this approach comes from increasing the number of categories, which also increases the likelihood that one or more categories would have a very low expected probability. Under these circumstances, of course, even adequately sampled categories may hold only a few individuals, thus increasing the influence of sampling error when weights are applied. To counter this tendency, we often recommend reducing the number of demographic categories by combining groups that are not expected to differ significantly in mean location on the ability variable. This practice can be applied to either marginal categories or joint cross-classifications when the latter are subject to the recoding procedure described in the previous paragraph.

### Limitations of the Study

This study evaluated only one method of post-stratification: raking with marginal probabilities as the input. We did not examine fully cross-classified post-stratification (i.e., a method that takes joint probabilities into account). Instead, we analyzed norm samples drawn from Population 5 (biased joint distributions) to determine the performance of raking under conditions where the marginal probabilities are representative, but the joint probabilities are not. In Population 5, we did not find that WCN, which includes raking, yielded increased norm-score bias compared with the benchmark condition. This result may have been due to the magnitude of non-representativeness in the cross-classification cells. The demographic deficiencies in these cells may not have been great enough to expose the inability of raking to compensate for such deficiencies.

In our study, we simulated three demographic variables (education, ethnicity, and region), with varying levels of correlation with the latent cognitive ability (strong, moderate, and weak, respectively). We did not model any interactions among these three variables. Demographic variables that interact in their effects on cognitive ability might yield larger disturbances in the raw score distributions of the cross-classification cells. Under these conditions, as we have demonstrated, weighting carries the risk of increasing norm-score bias. However, the main effect of education on test scores in our study probably represents the upper limit of analogous effects that could occur in real-world normative samples. With demographic variables that have smaller effect sizes, of course, we can expect resulting norm-score biases to also diminish in magnitude.

A second limitation was that our study modeled only one latent psychological variable: a cognitive ability that increases monotonically with increasing age. Other variables measured by psychometric tests (e.g., the “big five” personality traits, Donnellan & Lucas, 2008) may not manifest the same dependency on age, and they may be affected by demographic variables with different characteristics than the ones simulated in our study. When norming tests of personality traits, therefore, it may be appropriate to apply a weighting method that is not combined with continuous norming procedures.

A third caution relates to the mathematical underpinnings of the cNORM norming process. cNORM uses a semi-parametric continuous norming method that requires the expansion of a Taylor polynomial (for more details, see A. Lenhard et al., 2018b). The modeling process calls for the specification of a parameter (*k)* that sets an upper bound on the exponents of person location and age. In the current study, we used a default value of *k* = 4 for both location and age. It is possible that more precise models of the latent cognitive ability could have been obtained with different values of *k*. Simulation studies published elsewhere (Gary & Lenhard, 2021; A. Lenhard et al., 2019; W. Lenhard & Lenhard, 2021) have compared norm-score bias across a range of values of *k*. These findings suggest that *k* = 5 for location and *k* = 3 for age provide an optimal balance between norm score accuracy and processing load. As a result, we have selected these values as the defaults for the current version of cNORM.

Finally, our study examined weighting only as applied to the semi-parametric continuous norming method implemented in the cNORM package. We did not combine weighting with other continuous norming approaches, such as parametric continuous norming (e.g., Stasinopoulos et al., 2018) nor did we combine it with traditional raw-to-norm-score mapping performed separately per age group. The latter does not seem very promising anyway, since traditional approaches usually lead to norm-score bias that is more than three times as high on average as compared with continuous norming with the same sample size, when applied on perfectly representative samples (W. Lenhard & Lenhard, 2021). Therefore, traditional norming would have to benefit much more from weighting than continuous norming to overcome this general shortfall. There is simply no reason why such a disproportionate benefit should actually occur. Raking per age group—which would be necessary when combined with traditional norming approaches—might even entail its own pitfalls: The smaller the sample, the higher the expected deviations from representativeness. But as we have shown in this study, large deviations from representativeness can in some cases lead to suboptimal results of weighting techniques. To put it in a nutshell: Continuous norming is a major advance over traditional norming methods and WCN is still one step further.

Concerning parametric continuous norming approaches, we earlier pointed out in this article that the semi-parametric continuous method, because it does not rely on splines to model age-related changes in ability, may be better suited for certain conditions of non-representativeness in normative samples (e.g., the clustered distributions modeled in population 6). Moreover, we have demonstrated elsewhere (see A. Lenhard et al., 2019) that the cNORM approach yields less norm-score bias than parametric continuous norming with skewed raw score distributions and with sample sizes of 150 or less per age group. Yet, the efficiency of post-stratification techniques combined with parametric continuous norming remains to be investigated, as other regression-based norming methods (e.g., Oosterhuis, 2017) and parametric continuous norming approaches (e.g., Voncken et al., 2019) might also benefit from the use of weighing methods. Please note that the sample weighting itself starts before the actual norming process begins.

The application of weighting techniques to the norming of psychometric tests is a relatively new area of study. Unsurprisingly, therefore, several additional research questions emerged from the current simulation protocol. For example, we implemented raking weights twice within cNORM: Once during the ranking of raw scores and then again during regression modeling. But we did not evaluate the relative value in terms of reducing norm-score error of the second step. It is therefore possible that applying weights to the regression analysis was of little benefit or that it may have even increased norm-score bias. The latter might occur because, as noted previously, weighting can multiply the effects of sampling error in underrepresented groups.

## Concluding Remarks and Outlook

Weighting techniques are no substitute for the painstaking process of assembling a demographically representative normative sample. Our study has shown, however, that if such samples still exhibit reasonably small departures from representativeness, the weighting methods implemented in cNORM offer a useful way of mitigating any resulting norm score bias.

## Notes

1. For simplicity’s sake, we assume at this point that the number of individuals who identify as nonbinary is negligible.

2. *SD* is constrained to 1 across all cells of Table 2.
